# The Role of Sleep Disturbances in Alzheimer's Disease Progression: A Systematic Review

**DOI:** 10.7759/cureus.86450

**Published:** 2025-06-20

**Authors:** Abdallah Faisal Hassan Mohmmed Ahmed, Nour Mohamed Ahmed Emam Mohamed Ahmed, Ayman Abass Abdulwahid Elsamany, Hosam Eldin Makki Mohammed Mohammadany, Sara Abdalaziz Mohammed Ibrahim, Fatima Ahmed Mohamed Mustafa, Mustafa Mansour Mohammed Mustafa

**Affiliations:** 1 Neurology, Mubarak Al-Kabeer Hospital, Jabriya, KWT; 2 Internal Medicine, University of Gezira, Wad Madani, SDN; 3 Internal Medicine, Prince Mohammed Bin Abdulaziz Hospital, National Guard - Health Affairs, Medina, SAU; 4 Internal Medicine, Ibri Regional Hospital, Ibri, OMN; 5 Internal Medicine, Sheikh Jaber Al-Ahmad Al-Sabah Hospital, South Surra, KWT; 6 Internal Medicine, Dana Medical Center, HLMG Healthline Medical Group, Abu Dhabi, ARE; 7 Emergency Department, Port Sudan Judiciary Clinic, Red Sea State, SDN; 8 Internal Medicine, Mubarak Al-Kabeer Hospital, Jabriya, KWT

**Keywords:** alzheimer’s disease, biomarkers, cognitive decline, obstructive sleep apnea, rem sleep, sleep disturbances

## Abstract

Sleep disturbances are increasingly recognized as potential contributors to Alzheimer’s disease (AD) progression, but their exact role remains debated. This systematic review aims to synthesize existing evidence on the association between sleep disturbances, particularly disruptions in sleep architecture and obstructive sleep apnea (OSA), and cognitive decline or AD biomarkers, while exploring underlying mechanisms. Following Preferred Reporting Items for Systematic Reviews and Meta-Analyses (PRISMA) guidelines, 14 studies were selected from a comprehensive search of PubMed, Excerpta Medica Database (Embase), Psychological Information Database (PsycINFO), and Web of Science. Eligible studies included observational and longitudinal designs assessing sleep disturbances and their links to cognitive outcomes or AD pathology. Risk of bias was evaluated using the Newcastle-Ottawa Scale (NOS). Disruptions in rapid eye movement (REM) and non-rapid eye movement (NREM) sleep, such as reduced REM duration and fragmented sleep, were consistently associated with cognitive decline and new-onset dementia. Severe OSA increased AD risk, with hypoxia and sleep fragmentation implicated in neurodegeneration. Both objective (e.g., actigraphy) and subjective sleep measures predicted cognitive impairment, while cerebrospinal fluid (CSF) biomarkers and apolipoprotein E epsilon 4 (APOE ε4) genotype moderated these associations. Mechanistically, sleep disturbances may impair glymphatic clearance and exacerbate amyloid-β accumulation. Sleep disturbances, particularly REM/NREM disruptions and OSA, are robustly linked to AD progression, suggesting their potential as early biomarkers or therapeutic targets. However, causal inferences are limited by observational designs. Future research should prioritize interventional studies to determine whether improving sleep can mitigate AD risk.

## Introduction and background

Alzheimer’s disease (AD), the most common form of dementia, is a progressive neurodegenerative disorder characterized by cognitive decline, memory loss, and functional impairment. With an aging global population, the prevalence of AD is projected to triple by 2050, posing a significant public health and economic burden [1]. While the exact etiology of AD remains incompletely understood, emerging evidence suggests that sleep disturbances may play a critical role in its pathogenesis and progression. Sleep, a fundamental biological process, is essential for cognitive function, synaptic plasticity, and the clearance of neurotoxic proteins such as beta-amyloid (Aβ) and tau [2]. Disruptions in sleep architecture, particularly in rapid eye movement (REM) and non-REM (NREM) sleep, have been implicated in accelerating neurodegeneration [3], yet the precise mechanisms and clinical implications of this relationship remain an area of active investigation. Pioneering work by Phyllis Zee and colleagues has been instrumental in transforming our understanding of how circadian rhythm disruptions and poor sleep quality contribute to AD development, highlighting the importance of sleep regulation in neurodegenerative risk [1].

Growing epidemiological and neurobiological research highlights a bidirectional link between sleep disturbances and AD [4]. On one hand, sleep fragmentation, insomnia, and obstructive sleep apnea (OSA) may contribute to AD pathology by impairing glymphatic clearance, promoting neuroinflammation, and exacerbating amyloid and tau accumulation [5]. On the other hand, early AD-related neurodegeneration in sleep-regulating brain regions, such as the brainstem and hypothalamus, may itself disrupt sleep-wake cycles [6]. This complex interplay suggests that sleep disturbances could serve as both a risk factor and an early biomarker of AD. However, inconsistencies persist across studies regarding which specific sleep parameters, such as sleep duration, sleep efficiency (the proportion of time spent asleep while in bed), or OSA severity, are most strongly associated with cognitive decline. Additionally, the extent to which sleep interventions could mitigate AD risk remains uncertain.

To address these gaps, this systematic review synthesizes evidence from 14 studies examining the relationship between sleep disturbances and AD progression. We focus on key sleep metrics, including polysomnography (a comprehensive sleep study that records brain waves, oxygen levels, heart rate, and breathing), actigraphy (a wearable device that tracks movement to estimate sleep patterns), and OSA-related hypoxia, and their associations with cognitive decline, incident dementia, and AD biomarkers. By evaluating prospective and retrospective observational studies, this review aims to clarify whether sleep disturbances are a modifiable risk factor for AD or a secondary consequence of neurodegeneration. Furthermore, we explore potential mechanisms underlying these associations, such as impaired Aβ clearance, synaptic dysfunction, and circadian rhythm disruption.

Given the urgent need for early intervention strategies in AD, understanding the role of sleep in disease progression has significant clinical implications. If sleep disturbances indeed contribute to AD pathogenesis, then sleep optimization through behavioral, pharmacological, or OSA treatment approaches could represent a promising therapeutic avenue. Conversely, if sleep disruptions are primarily a downstream effect of neurodegeneration, they may still serve as valuable diagnostic or prognostic markers. By critically appraising the existing literature, this review seeks to inform future research directions and guide clinical practice in managing sleep and cognitive health in aging populations.

## Review

Methodology

This systematic review was conducted following the Preferred Reporting Items for Systematic Reviews and Meta-Analyses (PRISMA) guidelines to ensure methodological rigor and transparency [7]. The objective was to synthesize existing evidence on the association between sleep disturbances and AD progression, focusing on observational and longitudinal studies that examined how disruptions in sleep architecture, obstructive sleep apnea, and other sleep disorders influence cognitive decline and AD risk. 

*Eligibility Criteria* 

Studies were included if they investigated the relationship between sleep disturbances, such as sleep fragmentation, obstructive sleep apnea, or abnormalities in REM/NREM sleep, and cognitive decline or AD progression in human participants. Eligible study designs comprised cohort studies, case-control studies, and cross-sectional studies with clearly defined outcomes related to AD or cognitive impairment. Exclusion criteria were applied to animal studies, review articles, conference abstracts, and studies that did not report cognitive or AD-related outcomes. Only peer-reviewed articles published in English were considered to ensure consistency in data interpretation. 

Search Strategy and Information Sources

A comprehensive literature search was conducted across multiple electronic databases, including PubMed, Excerpta Medica Database (Embase), Psychological Information Database (PsycINFO), and Web of Science, from their inception to the most recent available data. We did not use any publication data restriction to cover all the available literature relevant to this systematic review. The search strategy incorporated a combination of controlled vocabulary terms and keywords related to sleep disorders and AD. To minimize the risk of missing relevant studies, additional manual searches were performed by reviewing the reference lists of included articles and key review papers in the field. The detailed Search Strategy for each database is provided in the Appendices section of this review.

Study Selection Process

The study selection process involved a two-stage screening approach to ensure methodological consistency. First, two independent reviewers screened titles and abstracts to identify potentially relevant studies based on the predefined eligibility criteria. Disagreements were resolved through discussion or consultation with a third reviewer. In the second stage, full-text articles of the selected studies were assessed for eligibility. Studies that met all inclusion criteria were retained for data extraction, while those excluded were documented with justification. A PRISMA flow diagram was utilized to illustrate the selection process, detailing the number of records identified, screened, and included, along with reasons for exclusion at each stage. 

Data Extraction and Synthesis

A standardized data extraction form was developed to systematically collect key information from each study. Extracted data included study characteristics such as author, year, country, and study design; participant demographics including age, sex, and baseline cognitive status; details on sleep disturbance assessment methods; and cognitive or AD-related outcomes. Findings were synthesized narratively due to the heterogeneity in study designs, sleep measurement tools, and outcome definitions. Studies were grouped thematically based on the type of sleep disturbance investigated and their reported associations with cognitive decline or AD biomarkers. 

*Risk of Bias Assessment* 

The methodological quality of the included studies was assessed using the Newcastle-Ottawa Scale (NOS) risk of bias tool [8], which evaluates three domains: selection of study groups, comparability of groups, and ascertainment of outcomes. Cross-sectional studies were appraised using an adapted version of the NOS. Each study was rated on a star-based system, with higher scores indicating lower risk of bias. Studies were categorized as having low, moderate, or high risk of bias based on their total scores.

Data Analysis and Reporting

Given the heterogeneity in study methodologies and outcome measures, conducting a meta-analysis was not appropriate. Therefore, a rigorous qualitative synthesis was performed, guided by established narrative synthesis methods to enhance transparency and reproducibility. Patterns and consistencies across studies were identified through systematic coding and thematic analysis, following recommendations for qualitative evidence synthesis in systematic reviews. To ensure the validity of this approach, two independent reviewers extracted and cross-verified the key findings, and any discrepancies were resolved through discussion or consultation with a third reviewer. Recurring themes identified included the impact of reduced REM sleep, the contribution of OSA to dementia risk, and the link between poor sleep efficiency and cognitive decline. The synthesis also accounted for potential confounding factors such as age, genetic predisposition, and comorbidities. The results were presented in a structured narrative format, clearly outlining the strength of evidence and highlighting gaps that warrant further research. 

Results

Study Selection Process

**Figure 1:**
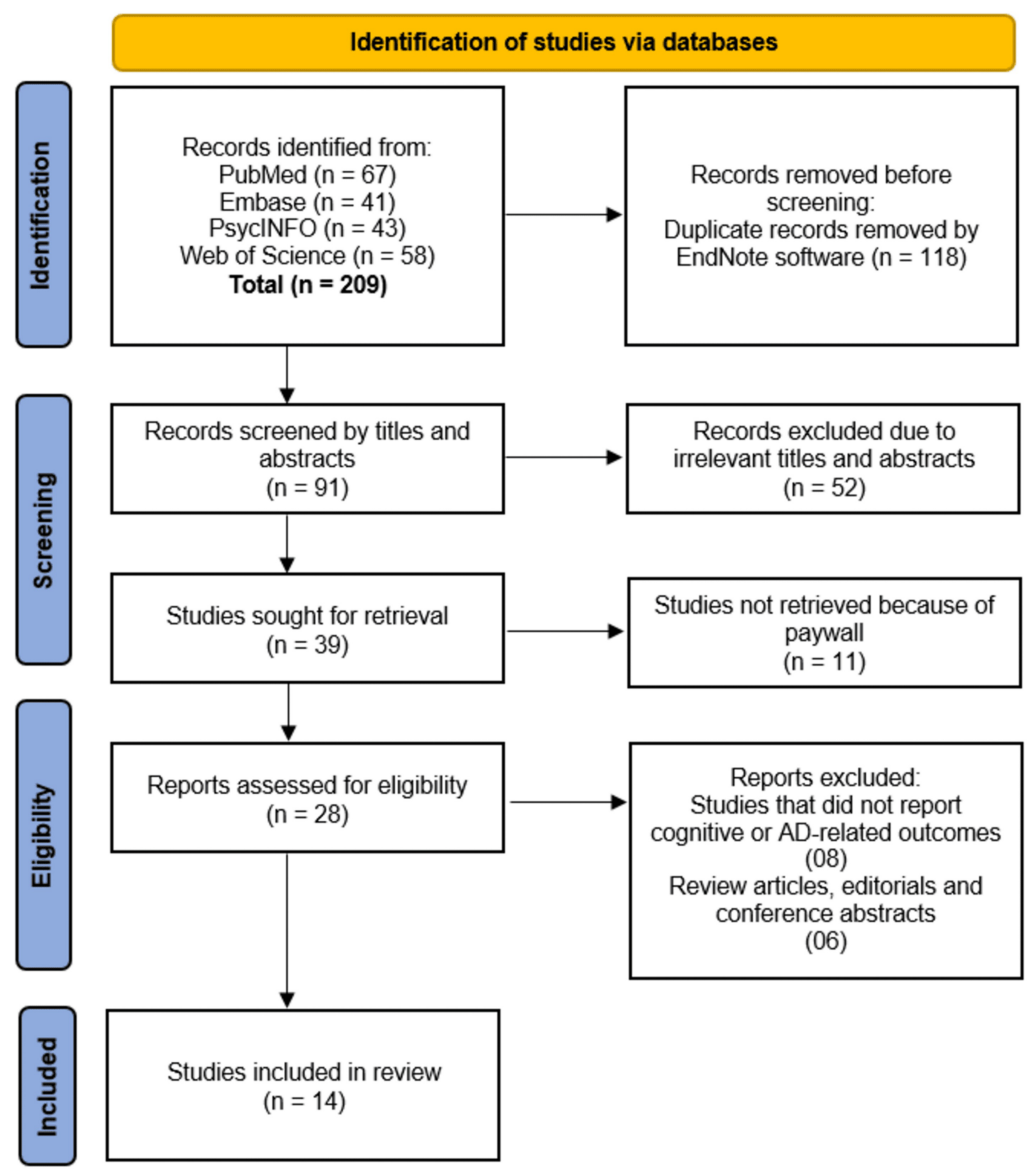
Visual Representation of Studies Selection Process on PRISMA Flowchart PRISMA: Preferred Reporting Items for Systematic Reviews and Meta-Analyses

The initial search across PubMed, Embase, PsycINFO, and Web of Science yielded 209 records, from which 118 duplicates were removed. The remaining 91 records were screened by title and abstract, leading to the exclusion of 52 irrelevant studies. Of the 39 full-text articles sought for retrieval, 11 were unavailable due to paywall restrictions. The remaining 28 reports were assessed for eligibility, with 14 excluded for not reporting cognitive or AD-related outcomes (n = 8) or being review articles, editorials, or conference abstracts (n = 6). Ultimately, 14 studies met the inclusion criteria and were incorporated into the review (Figure 1).

Characteristics of Included Studies

This review included 14 studies [9-22] investigating the relationship between sleep disturbances and AD progression. The studies were conducted across diverse populations, including community-dwelling older adults, individuals with mild cognitive impairment (MCI), and preclinical AD cohorts. Key study characteristics are summarized in Table 1. The sample sizes ranged from 61 to 9,066 participants, with follow-up durations varying from two to 19 years. Most studies employed prospective cohort designs [9,11,14], while others utilized observational or case-control methodologies [18,20]. Sleep disturbances were assessed using polysomnography, actigraphy, and self-reported measures, with outcomes focusing on cognitive decline, incident dementia, or AD biomarkers.

**Table 1:** Characteristics of Included Studies AD: Alzheimer’s disease; MCI: mild cognitive impairment, aMCI; amnestic mild cognitive impairment; NREM: non-rapid eye movement; REM: rapid eye movement; PSG: polysomnography; OSA: obstructive sleep apnea; AHI: Apnea-Hypopnea Index; EEG: electroencephalography; MRI: magnetic resonance imaging; MoCA-SA: Montreal Cognitive Assessment – Survey Adaptation; ARIC: Atherosclerosis Risk in Communities; CSF: cerebrospinal fluid; Aβ42: amyloid-beta 42; MMSE; Mini-Mental State Examination; 3MS: Modified Mini-Mental State Examination; TST: total sleep time; WASO: wake after sleep onset; SE: sleep efficiency; LWEP: long wake episodes; PSQI: Pittsburgh Sleep Quality Index; ESS: Epworth Sleepiness Scale; ODI: Oxygen Desaturation Index; CAP: cyclic alternating pattern; Trails B: Trail Making Test Part B; SDB: sleep-disordered breathing

Author(s), Year	Country	Study Design	Sample Size	Participant Characteristics	Type of Sleep Disturbance Assessed	Assessment Tools/Methods	AD Stage	Key Findings
Suh et al., 2019 [9]	South Korea	Prospective Cohort	235	Community-dwelling elderly, cognitively normal at baseline; mean age 68 (SD 5) years; 60% female	Disruption in NREM/REM sleep architecture	Overnight polysomnography; analysis of NREM/REM sleep cycles and durations	Baseline: Cognitively normal; Follow-up: Development of MCI/dementia tracked over 4 years	Shorter average NREM/REM cycle length and shorter average REM duration per cycle were significantly associated with development of MCI/dementia
Song et al., 2015 [10]	United States	Population-based prospective cohort study	2,601	Community-dwelling men aged ≥67 years, free of probable dementia at baseline	Alterations in sleep stage distribution (Stage N1, REM, N2, N3)	In-home polysomnography; Cognitive outcomes assessed via Trail Making Test Part B and Modified Mini-Mental State Examination (3MS)	Not specified (participants were free of probable dementia at baseline)	Greater time in Stage N1 sleep and reduced time in REM sleep were significantly associated with increased cognitive decline over ~3.4 years; no significant association found for N2 and N3 stages
Pase et al., 2017 [11]	United States	Prospective cohort study	321	Community-based participants aged >60 years (mean age 67 ± 5 years; 50% male)	Sleep architecture (REM and non-REM sleep disturbances)	Home-based polysomnography; longitudinal follow-up (mean 12 ± 5 years)	At-risk for AD; 24 out of 32 incident dementia cases were Alzheimer’s type	Lower REM sleep % and longer REM sleep latency were associated with higher dementia risk; non-REM sleep stages not associated with risk
Menon et al., 2019 [12]	India	Prospective observational cohort study	61 (37 aMCI, 24 controls)	Subjects with aMCI and cognitively normal controls	OSA, sleep efficiency, REM duration	Standardized neuropsychological tests, sleep questionnaires, overnight PSG, multimodality MRI	aMCI; 3-year follow-up to monitor progression to AD	Occult moderate-to-severe OSA was more prevalent in aMCI (43.6%) vs. controls (22.7%). Higher AHI and apnea-hypopnea times were noted in aMCI. Better sleep efficiency and longer REM sleep were associated with better memory performance. Only 5% of aMCI subjects progressed to AD over 3 years, and sleep parameters did not predict progression.
McSorley et al., 2019 [13]	United States	Observational cohort	759	Older adults, nationally representative cohort (2010–2015)	Sleep disruption (wake after sleep onset, fragmentation, % sleep, wake bouts)	Actigraphy for objective sleep measures; self-report; MoCA-SA	NR	Actigraphic sleep disruption was associated with worse cognition cross-sectionally and with 5-year cognitive decline. Self-reported sleep showed little association with cognition. Associations stronger in men than women.
Lysen et al., 2020 [14]	Netherlands	Prospective Cohort	1322	Non-demented adults, mean age 66 ± 8 years, 53% women	Sleep latency, wake after sleep onset, time in bed, sleep efficiency, "lights out" time	Actigraphy	Incident AD during follow-up (not baseline)	Poor sleep parameters associated with increased risk of dementia, especially AD. No link found between 24-hour activity rhythm disturbances and dementia risk.
Lutsey et al., 2018 [15]	United States	Prospective cohort study	1,667	Middle-aged adults from the ARIC Study	OSA, short and long sleep duration	In-home PSG (1996–1998); dementia diagnosis from hospitalization codes (1996–2012) and neurocognitive exams with adjudication (2011–2013)	NR	Severe OSA was associated with increased risk of all-cause and Alzheimer's dementia. Sleeping <7 hours associated with increased risk of all-cause dementia.
Lutsey et al., 2016 [16]	United States	Prospective cohort study	966	Mean age 61 years; 55% women; community-based sample from ARIC study	OSA, hypoxemia, disordered breathing, sleep fragmentation, sleep duration	In-home PSG (1996–1998); Cognitive tests: Delayed Word Recall, Word Fluency, Digit Symbol Substitution, plus 10 additional tests (2011–2013); Multivariable linear regression	NR	No significant association between midlife sleep disturbances (including OSA) and cognitive decline over 15 years
Lucey et al., 2021 [17]	United States	Observational longitudinal study	100	Individuals undergoing standardized cognitive testing, APOE genotyping, and CSF biomarker analysis (preclinical to early AD)	Total sleep time, NREM/REM sleep, sleep efficiency, NREM slow wave activity	Sleep-wake activity monitoring over 4–6 nights; cognitive tests: Free and Cued Selective Reminding, Logical Memory Delayed Recall, Digit Symbol Substitution, MMSE; CSF biomarkers (tau, Aβ42)	Preclinical to early symptomatic AD	Non-linear relationship between sleep parameters and cognitive decline; both low and high extremes of sleep metrics were associated with cognitive decline, while mid-range values were linked to cognitive stability.
Djonlagic et al., 2019 [18]	United States	Nested case-control study	170 (85 cases, 85 controls)	Cognitively normal elderly women (mean age 83) at baseline, MMSE > 24	Sleep EEG abnormalities (quantitative changes)	PSG, quantitative EEG analysis (power density across bands: alpha, theta, sigma; NREM and REM sleep)	Preclinical (prior to MCI or dementia diagnosis)	Higher EEG power in alpha and theta bands (NREM) and alpha and sigma bands (REM) predicted progression to MCI or dementia. Traditional sleep measures did not show group differences.
Diem et al., 2016 [19]	United States	Prospective cohort study	1,245	Older adult women (mean age: 82.6 years) without dementia	Sleep efficiency, sleep latency, total sleep time variability	Actigraphy	NR	Low sleep efficiency (<74%), longer sleep latency, and high variability in sleep efficiency/total sleep time were associated with increased risk of MCI or dementia over ~4.9 years.
Carnicelli et al., 2019 [20]	Italy	Observational longitudinal study	30 total (19 with aMCI, 11 healthy controls)	Amnestic MCI patients (mean age 68.5 ± 7.0 years); healthy controls (mean age 69.2 ± 12.6 years)	Sleep architecture disruption, CAP changes	Ambulatory PSG; CAP analysis	aMCI, with follow-up classification into converters to Alzheimer’s Disease (N=11) and non-converters	MCI patients showed decreased REM sleep, CAP rate, and A1 index vs. controls. Among MCI, converters to AD had lower CAP rate, A1, and A3 indices than non-converters. Refined sleep metrics may predict progression to AD.
Blackwell et al., 2015 [21]	United States	Population-based longitudinal study	2,636	Community-dwelling older men (mean age 76.0 ± 5.3) without probable mild cognitive impairment or dementia	SDB including nocturnal hypoxemia, ODI, and AHI	In-home PSG; Modified Mini-Mental State Examination (3MS); Trails B; linear mixed models	NR	Nocturnal hypoxemia and ODI were modestly associated with greater global cognitive decline on 3MS; AHI was not significantly associated; no link with Trails B
Blackwell et al., 2014 [22]	United States	Population-based longitudinal study	2,822	Cognitively intact, community-dwelling older men (mean age 76 ± 5.3 years)	Objective: WASO, SE, LWEP; Subjective: Sleep quality, Daytime sleepiness, Total Sleep Time	Objective: Wrist actigraphy (TST, SE, WASO, LWEP); Subjective: PSQI, ESS, self-reported TST	Participants were cognitively intact at baseline	Poor sleep quality (low SE, high WASO, many LWEP) and poor self-reported sleep were associated with a 1.4–1.5× increased odds of clinically significant cognitive decline (e.g., 3MS, Trails B test); PSQI predicted decline in Trails B performance over time.

Sleep Architecture and Cognitive Decline

Disruptions in sleep architecture, particularly involving REM and NREM sleep, were commonly associated with cognitive decline in several studies; however, this relationship was not entirely consistent across all included studies. For example, Suh et al. found that shorter NREM/REM cycle duration and reduced REM sleep were significantly linked to the development of MCI or dementia over four years [9]. Similarly, Song et al. reported that increased Stage N1 sleep and reduced REM sleep were associated with greater cognitive decline, as measured by the Trail Making Test Part B and Modified Mini-Mental State Examination (3MS) [10]. Pase et al. observed that each 1% reduction in REM sleep increased the risk of incident dementia by 9%, suggesting a dose-dependent relationship [11]. However, other studies within the review did not find a significant association between REM sleep disturbances and cognitive impairment, indicating variability in this finding. These inconsistencies may be attributed to differences in study design, sample size, population characteristics, and methods of sleep assessment.

OSA and Dementia Risk

OSA and related hypoxemia emerged as significant risk factors for AD and all-cause dementia. Lutsey et al. demonstrated that severe OSA was associated with a 2.35-fold increased risk of all-cause dementia and a 1.66-fold risk of AD [15]. Menon et al. noted that OSA was more prevalent in aMCI patients (43.6%) compared to controls (22.7%), and higher AHI scores correlated with poorer visual memory [12]. However, Lutsey et al. found no association between midlife OSA and cognitive decline over 15 years, highlighting potential differences in the timing of OSA effects or any relationship at all [16].

Sleep Disruption and Biomarkers of AD

Sleep disturbances were linked to AD biomarkers in preclinical and early-stage populations. Lucey et al. reported a U-shaped relationship between sleep parameters (e.g., duration, efficiency) and cognitive decline, with both short and long sleep durations associated with worse outcomes [17]. The presence of CSF biomarkers (such as elevated tau and decreased Aβ42) and the APOE ε4 genotype were found to further moderate these associations, amplifying the relationship between sleep disturbances and cognitive decline. Djonlagic et al. identified quantitative EEG changes during NREM and REM sleep as predictors of MCI or dementia progression, suggesting early neural alterations [18].

Subjective and Objective Sleep Measures

Both subjective and objective sleep measures were consistently associated with cognitive outcomes across multiple studies, many of which used validated tools or formal sleep assessments. Blackwell et al. found that poor sleep efficiency, frequent awakenings, and self-reported poor sleep quality were associated with clinically significant cognitive decline over 3.4 years, using both self-reported questionnaires and actigraphy [22]. McSorley et al. reported that actigraphy-measured sleep disruption (e.g., wake after sleep onset) was cross-sectionally linked to lower cognitive scores and predicted five-year decline [13]. Diem et al. demonstrated that objectively measured low sleep efficiency (<74%) and irregular sleep patterns, assessed through actigraphy, significantly increased the risk of MCI or dementia in older women (Table 2) [19].

**Table 2:** Sleep Disturbance Effects on Alzheimer's Disease Progression NREM: non-rapid eye movement; REM: rapid eye movement; NR: not reported; MCI: mild cognitive impairment; 3MS: Modified Mini-Mental State Examination; OSA: obstructive sleep apnea; AHI: Apnea–Hypopnea Index; MoCA-SA: Montreal Cognitive Assessment – Survey Adaptation; CAP: cyclic alternating pattern; EEG: eectroencephalography; CSF: cerebrospinal fluid; APOE ε4: apolipoprotein E epsilon 4 genotype; SDB: sleep-disordered breathing; ODI: Oxygen Desaturation Index; SaO₂: arterial oxygen saturation; TST: total sleep time; PSQI: Pittsburgh Sleep Quality Index; ESS: Epworth Sleepiness Scale.

Author(s), Year	Sleep Disturbance Type	Mechanism Explored	Biomarkers Assessed	Cognitive Outcomes	Follow-Up Duration
Suh et al., 2019 [9]	Short NREM/REM cycle length and reduced REM sleep per cycle	Disruption in sleep architecture affecting memory consolidation and brain clearance during REM/NREM cycling	NR	Development of MCI or dementia	4 years
Song et al., 2015 [10]	Increased Stage N1 sleep and reduced REM sleep	Altered sleep architecture affecting cognitive performance	NR	Decline in Trail Making Test Part B and 3MS scores; higher Stage N1 linked with 2x greater cognitive decline; lower REM linked with more 3MS decline	3.4 years
Pase et al., 2017 [11]	Reduced REM sleep & prolonged REM latency	Disruption in REM sleep architecture affecting memory consolidation and neurodegeneration	NR	Increased risk of incident dementia; 9% increase per 1% reduction in REM sleep	Mean: 12 ± 5 years (max: 19 years)
Menon et al., 2019 [12]	OSA	Association between sleep-related breathing disturbances (e.g., apnea-hypopnea index) and sleep architecture (e.g., REM sleep, sleep efficiency) on cognition	NR	Better sleep efficiency and longer REM sleep correlated with improved associative learning and memory; higher AHI correlated with poorer visual memory	3 years
McSorley et al., 2019 [13]	Restricted Sleep Duration/Sleep Disruption (wake after sleep onset, fragmentation, % sleep, wake bouts)	Sleep disruption leading to cognitive decline	Actigraph-measured sleep parameters (wake after sleep onset, fragmentation, percentage sleep, wake bouts); Self-reported sleep characteristics	Worse cognition cross-sectionally (lower MoCA-SA scores); Increased odds of 5-year cognitive decline (≥4 points drop in MoCA-SA)	5 years
Lysen et al., 2020 [14]	Poor sleep: longer sleep latency, wake after sleep onset, longer time in bed, lower sleep efficiency, earlier lights out time	Association of sleep disruption with increased risk of dementia, particularly AD	Actigraphy data used as objective measure	Increased risk of dementia, specifically AD	Up to 11.2 years
Lutsey et al., 2018 [15]	OSA and Short Sleep Duration (<7 hrs)	Possible link between sleep fragmentation, hypoxia, and impaired cognitive resilience contributing to neurodegeneration	NR	Severe OSA associated with increased risk of all-cause dementia (RR 2.35) and AD (RR 1.66); short sleep duration associated with increased risk of all-cause dementia (RR 2.00)	15 years
Lutsey et al., 2016 [16]	OSA, Sleep Fragmentation, Sleep Duration, Hypoxemia	Hypoxia, sleep fragmentation, poor sleep quality/quantity	NR	No association found between OSA/severity/sleep measures and cognitive decline across multiple cognitive tests	Median: 14.9 years (max: 17.3 years)
Lucey et al., 2021 [17]	Short and Long Sleep Duration; Low or High Non-REM and REM Sleep; Low Sleep Efficiency; Abnormal Slow Wave Activity	Non-linear effects of sleep parameters on cognitive trajectory in preclinical/early AD; Sleep optimization as potential stabilizer of cognition	CSF total tauCSF amyloid-β42CSF tau/amyloid-β42 ratioAPOE ε4 genotype	Decline in preclinical Alzheimer cognitive composite score at low and high values of sleep parameters; Stability of cognitive function at mid-range levels of sleep	Longitudinal (specific duration not given; cognitive testing done at each clinical visit)
Djonlagic et al., 2019 [18]	Quantitative EEG changes during sleep	Alterations in EEG power density during NREM and REM sleep may predate cognitive decline	Absolute and relative EEG power in alpha, theta (NREM), and alpha, sigma (REM) bands	Development of mild cognitive impairment or dementia	5 years
Diem et al., 2016 [19]	Low sleep efficiency, long sleep latency, variability in sleep efficiency and total sleep time	Impaired sleep continuity, delayed sleep initiation, irregular sleep patterns	NR	Increased odds of developing MCI or dementia (OR up to 1.53 for lowest sleep efficiency)	4.9 (±0.6) years
Carnicelli et al., 2019 [20]	Disrupted sleep architecture (↓ REM sleep, ↓ CAP rate, ↓ A1 & A3 indices)	Relationship between sleep microstructure and progression from amnestic MCI to AD	CAP rate, A1 index, A3 index, REM sleep percentage	Conversion from amnestic mild cognitive impairment to AD	2 years
Blackwell et al., 2015 [21]	SDB, including nocturnal hypoxemia and ODI	Intermittent hypoxemia during sleep leading to reduced oxygenation	Oxygen saturation (SaO₂ <90%), ODI, AHI	Greater decline in global cognition (3MS); no significant effect on executive function (Trails B)	3.4 ± 0.5 years
Blackwell et al., 2014 [22]	Reduced sleep efficiency, greater wake after sleep onset, number of long wake episodes, poor self-reported sleep quality	Sleep fragmentation and poor sleep quality leading to cognitive decline	Objective wrist actigraphy measures (total sleep time, sleep efficiency, wake after sleep onset, long wake episodes), PSQI, ESS	Clinically significant decline on Modified Mini-Mental State Exam and Trails B test (executive function)	Mean 3.4 ± 0.5 years

Risk of Bias Findings

The methodological quality assessment revealed that seven studies [9-11,14-16,21,22] achieved the maximum score of 9 stars, indicating low risk of bias across selection (4 stars), comparability (2 stars), and outcome (3 stars) domains. These studies predominantly employed prospective or population-based cohort designs with rigorous control for confounders and validated outcome measures. In contrast, six studies [12,13,17-20] scored 6 stars, reflecting moderate risk of bias due to limitations in comparability (1 star) or outcome assessment (2 stars), often associated with observational or longitudinal designs with less stringent control for covariates. No studies were rated as high risk of bias. The consistent low-risk ratings among prospective cohort studies strengthen confidence in their findings, while the moderate-risk studies highlight the need for cautious interpretation of results where residual confounding or measurement biases may persist (Table 3). 

**Table 3:** Risk of Bias Results for all Included Studies Using NOS risk of Bias Tool NOS: Newcastle–Ottawa scale

Author(s), Year	Study Design	Selection (Max 4)	Comparability (Max 2)	Outcome (Max 3)	Total Stars (Max 9)	Risk of Bias
Suh et al., 2019 [9]	Prospective Cohort	4	2	3	9	Low
Song et al., 2015 [10]	Population-based Cohort	4	2	3	9	Low
Pase et al., 2017 [11]	Prospective Cohort	4	2	3	9	Low
Menon et al., 2019 [12]	Observational Cohort	3	1	2	6	Moderate
McSorley et al., 2019 [13]	Observational Cohort	3	1	2	6	Moderate
Lysen et al., 2020 [14]	Prospective Cohort	4	2	3	9	Low
Lutsey et al., 2018 [15]	Prospective Cohort	4	2	3	9	Low
Lutsey et al., 2016 [16]	Prospective Cohort	4	2	3	9	Low
Lucey et al., 2021 [17]	Longitudinal Observational	3	1	2	6	Moderate
Djonlagic et al., 2019 [18]	Nested Case-Control	3	1	2	6	Moderate
Diem et al., 2016 [19]	Prospective Cohort	3	1	2	6	Moderate
Carnicelli et al., 2019 [20]	Longitudinal Observational	3	1	2	6	Moderate
Blackwell et al., 2015 [21]	Population-based Cohort	4	2	3	9	Low
Blackwell et al., 2014 [22]	Population-based Cohort	4	2	3	9	Low

Discussion

Sleep Architecture Findings

The findings of this systematic review underscore the significant role of sleep disturbances in the progression of AD and cognitive decline, as evidenced by the 14 included studies. Disruptions in sleep architecture, particularly involving REM and NREM sleep, are consistently associated with cognitive impairment and the development of AD. For instance, Suh et al. demonstrated that shorter NREM/REM cycle durations and reduced REM sleep were linked to the development of MCI or dementia over a four-year follow-up [9]. This aligns with existing literature suggesting that REM sleep is critical for memory consolidation and synaptic plasticity, and its disruption may impair these processes, thereby accelerating cognitive decline [23]. Similarly, Song et al. found that increased Stage N1 sleep and reduced REM sleep were associated with greater cognitive decline, as measured by the Trail Making Test Part B and the 3MS [10]. These findings support the hypothesis that fragmented sleep and reduced REM sleep may disrupt the clearance of neurotoxic proteins, such as beta-amyloid, which accumulate in AD [24]. Pase et al. further reported a dose-dependent relationship between reduced REM sleep and incident dementia, with each 1% reduction in REM sleep increasing the risk by 9% [11]. This suggests that REM sleep may serve as a protective factor against neurodegeneration, and its loss could be an early marker of AD progression.

OSA and Cognitive Outcomes

OSA emerged as another critical risk factor for AD and all-cause dementia, although studies showed mixed results depending on the timing of OSA assessment. Lutsey et al. found that severe OSA was associated with a 2.35-fold increased risk of all-cause dementia and a 1.66-fold risk of AD, emphasizing the role of intermittent hypoxia and sleep fragmentation in neurodegeneration [15]. Menon et al. noted a higher prevalence of OSA in amnestic MCI patients (43.6%) compared to controls (22.7%), with higher apnea-hypopnea index (AHI) scores correlating with poorer visual memory [12]. However, Lutsey et al. found no association between midlife OSA and cognitive decline over 15 years, suggesting that the timing of OSA may influence its impact on cognition [15]. This discrepancy may reflect differences in study populations or the progressive nature of OSA-related damage, which may take decades to manifest as cognitive decline. These findings align with previous research indicating that chronic hypoxia and oxidative stress from OSA can lead to neuronal injury and inflammation, both implicated in AD pathogenesis [25].

Mechanisms and Biomarkers

Mechanistic insights and biomarker findings were central to this review. Lucey et al. reported a U-shaped relationship between sleep parameters (e.g., duration, efficiency) and cognitive decline, with both short and long sleep durations linked to worse outcomes [17]. This suggests that optimal sleep duration and quality are crucial for cognitive health, and deviations in either direction may be harmful. The study also highlighted the moderating role of CSF biomarkers (e.g., tau, Aβ42) and APOE ε4 genotype, indicating that sleep disturbances may interact with genetic and molecular factors to accelerate AD progression. This aligns with evidence that sleep deprivation increases beta-amyloid accumulation in the brain [26]. Djonlagic et al. further identified quantitative EEG changes during NREM and REM sleep as predictors of MCI or dementia progression, suggesting that early neural alterations in sleep may precede clinical cognitive symptoms [18].

Carnicelli et al. found that disrupted sleep architecture, including reduced REM sleep and altered cyclic alternating pattern (CAP) metrics, predicted conversion from amnestic MCI to AD, implying that sleep microstructure may reflect early neurodegenerative changes [20]. Lysen et al. [14] reported that poor sleep parameters, such as longer sleep latency and lower sleep efficiency, were associated with increased dementia risk, particularly AD. Animal studies have shown that sleep deprivation impairs glymphatic clearance, resulting in the accumulation of neurotoxic proteins [27]. Blackwell et al. highlighted the role of hypoxia in OSA-related cognitive decline, finding that nocturnal hypoxemia, rather than AHI, was more strongly linked to cognitive deterioration [21]. Together, these findings illustrate multiple pathways through which sleep disruption may contribute to AD pathogenesis.

Subjective vs. Objective Measures

Both subjective and objective sleep measures were found to predict cognitive outcomes, though objective measures often provided stronger associations. Blackwell et al. showed that poor sleep efficiency, frequent awakenings, and self-reported poor sleep quality were linked to clinically significant cognitive decline over 3.4 years [22]. Similarly, McSorley et al. found that actigraphy-measured sleep disruption (e.g., wake after sleep onset) was associated with lower cognitive scores and predicted five-year decline [13]. Diem et al. further showed that low sleep efficiency (<74%) and sleep pattern variability increased the odds of MCI or dementia in older women [19]. These findings support the value of combining subjective reports with objective data to better understand sleep’s impact on cognition.

Implications and Future Directions

The heterogeneity in study designs, populations, and methodologies warrants consideration. Some studies focused on community-dwelling older adults [9], while others examined clinical populations, such as individuals with MCI [12]. Despite these differences, the consistency of findings across diverse cohorts strengthens the evidence for a causal link between sleep disturbances and AD progression. However, the lack of randomized controlled trials limits definitive causal inferences. Future research should test whether interventions targeting sleep can effectively mitigate cognitive decline and delay AD onset.

Limitations

This systematic review has several limitations. First, the included studies varied in their methodologies, including differences in sleep assessment tools (e.g., polysomnography, actigraphy, self-reports) and cognitive outcome measures, which may have introduced heterogeneity. Second, most studies were observational, precluding causal inferences. Third, the follow-up durations varied widely, from two to 19 years, which may have influenced the detection of long-term cognitive outcomes. Finally, the studies predominantly included older adults, limiting the generalizability of findings to younger populations or those with severe sleep disorders.

## Conclusions

Sleep disturbances, particularly disruptions in REM/NREM sleep architecture and OSA, are significantly associated with AD progression and cognitive decline. The findings suggest these disturbances may contribute to AD pathogenesis through impaired protein clearance, neuroinflammation, and hypoxia, while also serving as potential early biomarkers. Although the observational nature of most studies precludes definitive causal claims, the consistency of results across diverse populations underscores the importance of addressing sleep health in aging individuals. Future research should focus on longitudinal and interventional studies to establish causality and evaluate whether targeted sleep interventions could delay or prevent cognitive impairment, offering a promising non-pharmacological approach to reducing the global burden of AD.

## Appendices

**Table 4:** Search Strategy for Databases Used in this Study

Database	Search String
PubMed	("Alzheimer Disease"[Mesh] OR "Alzheimer*" OR "Dementia") AND ("Sleep Wake Disorders"[Mesh] OR "Sleep Disturbances" OR "Sleep Disorder*" OR "Sleep Fragmentation" OR "Insomnia" OR "Obstructive Sleep Apnea" OR "OSA" OR "REM sleep" OR "NREM sleep")
Embase	'alzheimer disease'/exp OR 'alzheimer*' OR 'dementia' AND ('sleep disorder'/exp OR 'sleep disturbance*' OR 'sleep fragmentation' OR 'insomnia' OR 'obstructive sleep apnea' OR 'osa' OR 'rem sleep' OR 'nrem sleep')
PsycINFO	DE "Alzheimer's Disease" OR TI ("Alzheimer*" OR "Dementia") AND DE "Sleep Disorders" OR ("Sleep Disturbance*" OR "Sleep Fragmentation" OR "Insomnia" OR "Obstructive Sleep Apnea" OR "OSA" OR "REM sleep" OR "NREM sleep")
Web of Science	TS=("Alzheimer*" OR "Dementia") AND TS=("Sleep Disturbance*" OR "Sleep Disorder*" OR "Sleep Fragmentation" OR "Insomnia" OR "Obstructive Sleep Apnea" OR "OSA" OR "REM sleep" OR "NREM sleep")
